# Effectiveness of surgical treatments on healing of cartilage and function level in patients with osteochondral lesions of the tibial plafond: A systematic review

**DOI:** 10.1016/j.jor.2021.08.011

**Published:** 2021-08-17

**Authors:** Eline M. Jagtenberg, Pishtiwan H.S. Kalmet, Maartje A.P. de Krom, Joris P.S. Hermus, Henk A.M. Seelen, Martijn Poeze

**Affiliations:** a Maastricht University Medical Centre, Dept. of Trauma Surgery, Maastricht, the Netherlands; b Maastricht University Medical Centre, Dept. of Orthopedic Surgery, Maastricht, the Netherlands; c Adelante Centre of Expertise in Rehabilitation and Audiology, Hoensbroek, the Netherlands; d Research School CAPHRI, Dept. of Rehabilitation Medicine, Maastricht University, Maastricht, the Netherlands; e Nutrim School for Nutrition, Toxicology and Metabolism, Maastricht University, Maastricht, the Netherlands

**Keywords:** Ankle, Osteochondral lesion, Defect, Tibial plafond, Systematic review, Arthroscopy

## Abstract

**Background:**

Osteochondral lesions of the tibial plafond (OLTPs) occur less frequently than those of the talus, and treatment guidelines have not been determined. The aim of the current review was to evaluate the effectiveness of surgical treatments on the healing of cartilage and on function level, i.e. pain reduction, reduced swelling and improved joint range of motion, in patients with OLTPs.

**Methods:**

A comprehensive literature search in PubMed/MEDLINE, Cochrane Database of Systematic Reviews and Google Scholar was performed up to December 2020. The outcome measures were healing of cartilage and function level.

**Results:**

Four studies investigating treatment of OLTPs were included. Three studies investigated treatment by means of microfracture. One of these studies showed an osteochondral defect filling in 52.0% of patients. All three studies showed an improvement in function level. Antegrade drilling was evaluated in one study, showing contrasting results in two patients. One-step bone marrow-derived cell transplantation was evaluated in one study, showing an osteochondral defect filling in 68.0% of patients and improvements in patients' function level.

**Conclusions:**

Arthroscopic treatment of OLTPs by means of microfracture and bone marrow-derived cell transplantation (BMDCT) seem effective for the outcome at the patient's function level, while BMDCT showed more promising results regarding defect filling. However, this is based on the current available evidence with poor quality of methodology. Further research is of paramount importance to understand this injury and to evaluate the best treatments.

## Abbreviations

AOFAS: the American Orthopaedic Foot and Ankle Society
BMDCT: bone marrow-derived cell transplantation
FAAM: the Foot and Ankle Ability Measure
FAAM ADL: the Foot and Ankle Ability Measure Activity Daily Living Subscale
FAOS: the Foot and Ankle Outcome Score
ICRS: the International Cartilage Repair Society Cartilage Lesion Classification System
MOCART: the Magnetic Resonance Observation of Cartilage Repair
MRI: Magnetic Resonance Imaging
NOS: Newcastle-Ottawa Scale
OCLs: osteochondral lesions
OLTPs: osteochondral lesions of the tibial plafond
OLTs: osteochondral lesions of the talus
PRISMA: Preferred Reporting Items for Systematic Reviews and Meta-Analyses
RCT: Randomized Controlled Trial
SF-12: Short-form Generic Measure of Health Status
VAS: Visual Analogue Scale

## 1 Introduction

In approximately 50% of acute ankle fractures and sprains, osteochondral lesions (OCLs) of the ankle may occur.^1^ OCLs are defined as damage to articular cartilage and its subchondral bone, causing deterioration in functional outcome, i.e. deep ankle pain, stiffness, ankle joint locking, swelling, and a limited range of motion.^2^ These symptoms result in a decrease in quality of life.^3^

While OCLs of the ankle most frequently concern the talus (OLTs), isolated osteochondral lesions of the tibia plafond (OLTPs) occur considerably less frequent.^4^ According to Mologne et al., 2.6% of patients with OCLs (i.e. 23/880) suffered from OLTPs in isolation.^5^ Although no clear explanation is available why OLTPs are less common than OLTs, it is suggested that the cartilage of the distal tibial plafond is less susceptible to damage due to its concave shape, greater articular cartilage stiffness and rich arterial supply.^4^^,^^6^, ^7^, ^8^ Due to the rarity of OLTPs, there is little consensus on its treatment.^9^^,^^10^

The initial treatment goal for OCLs of the ankle is to improve patients' outcome regarding their function level, i.e. to reduce pain and swelling, and improve joint range of motion.^11^ If left untreated, OCLs can lead to abnormal articular stress patterns, eventually leading to abnormal cartilage wear, cyst formation, cancellous bone remodeling and osteoarthritis.^5^^,^^12^ Zengerink et al.^11^ found that treatment of OLTs by bone marrow stimulation and debridement is the most effective, and therefore the same treatment may also be applicable in OLTPs.^9^ However, differences in composition of cartilage and accessibility of the lesions may result in different treatment outcomes.^3^

The aim of this systematic review is to evaluate the effectiveness of surgical treatments on the healing of cartilage and on function level, i.e. in terms of pain reduction, reduced swelling and improved joint range of motion, in patients with OLTPs.

## 2 Methods

### 2.1 Data sources and search strategy

This systematic review was written using the Preferred Reporting Items for Systematic Reviews and Meta-Analyses (PRISMA) guidelines.^13^^,^^14^

The PubMed/MEDLINE database and Cochrane Database of Systematic Reviews were screened up to December 2020. The following Medical Subject Headings (MeSH) terms and free terms were used: ((((surgical procedures, operative [MeSH Terms]) OR (treatment)) AND (((treatment outcome) OR (outcome)) OR (clinical outcome))) AND ((((tibial pilon) OR (tibia plafond)) OR (distal tibia)) OR (tibial plafond))) AND ((osteochondral lesion) OR (osteochondral injury)). No additional limits were applied. In addition, the first 100 entries of Google Scholar were searched, using the keywords: osteochondral lesion, osteochondral defect, tibial plafond, distal tibia, and therapy. Finally, reference lists of all included studies were manually reviewed.

### 2.2 Study selection

After removal of duplicates, manuscript title and abstract were screened regarding the inclusion criteria. Studies inclusion criteria were: 1) Randomized controlled trial (RCT), cohort studies (either prospective or retrospective) or quasi-experimental research evaluating the effectiveness of surgical treatment strategies for OLTP; 2) Full-text clinical studies in English; 3) A follow-up period of at least 1 year; 4) Description of healing of cartilage and/or effects of different treatment modalities on patients' function level (pain reduction, reduced swelling and improved joint range of motion). Exclusion criteria were: 1) Inadequate description of therapy; 2) Less than ten participants included; 3) No well-defined outcome reported; 4) Results not described per surgical treatment. For studies that potentially met eligibility criteria, full texts were obtained and screened to determine their final inclusion.

Search, screening and inclusion of eligible articles and data analysis of included articles were performed by two researchers (EJ and MK). In case of inter-observer disagreement, the study was discussed until consensus was reached.

### 2.3 Data extraction and methodological quality assessment

Study methodology information was collected, including design, number of patients included, and follow-up period. Patients' demographics were also collected, including age, gender, comorbidity, lesion type and size, and type of treatment. Furthermore, outcome parameters (healing of cartilage and function level, measured by scoring systems (i.e., MOCART, AOFAS, FAAM, FAOS, SF-12 and VAS score) were collected. The American Orthopaedic Foot and Ankle Society (AOFAS) score includes nine items divided into three subscales, i.e. pain, function and alignment, with a maximal score of 100 points indicating no symptoms of impairments.^15^ The Magnetic Resonance Observation of Cartilage Repair Tissue (MOCART) score consists of a set of variables to evaluate cartilage repair after treatment i.e. degree of defect filling, cartilage interface, surface, presence of adhesions, structure and signal intensity of the repair tissue, the subchondral lamina and bone, and the presence of effusion. A score is given to each variable, ranging from 0 to 100 points, in which a score of 100 points indicates the best cartilage healing.^16^ In the Foot and Ankle Ability Measure (FAAM), the Foot and Ankle Outcome Score (FAOS) and the Short-form Generic Measure of Health Status (SF-12) a higher score indicates a better function outcome, while in the Visual Analogue Scale (VAS), a higher score indicates that more pain is experienced.

Results are reported per treatment. Summary measures are presented qualitatively with a plus in case improvement after treatment was found. Healing of cartilage is presented in a table, showing the percentage of patients with a total osteochondral defect filling and the percentage of patients with subchondral edema or cysts.

Currently, no validated quality scores are available for case series, while in orthopedic literature the vast majority of studies concern case series study designs.^11^ Therefore, in the current systematic review an adjusted version of the Newcastle-Ottawa Scale (NOS),^17^ retrieved from the systematic review of Zengerink et al.,^11^ was used to differentiate between low or high risk of bias. The NOS was initially developed to assess the quality of non-randomized studies. However, since comparability and adjustment are not relevant to non-comparative studies like case series, these items were removed. This resulted in an adjusted NOS evaluating three items concerning study design, selection, and assessment of outcome.^18^ The adjusted NOS uses a “star” rating system. Two independent reviewers (EJ and MK) assessed the studies' risk of bias and reached consensus through discussion in case of disagreement.

## 3 Results

### 3.1 Study retrieval and characteristics

**Fig. 1:**
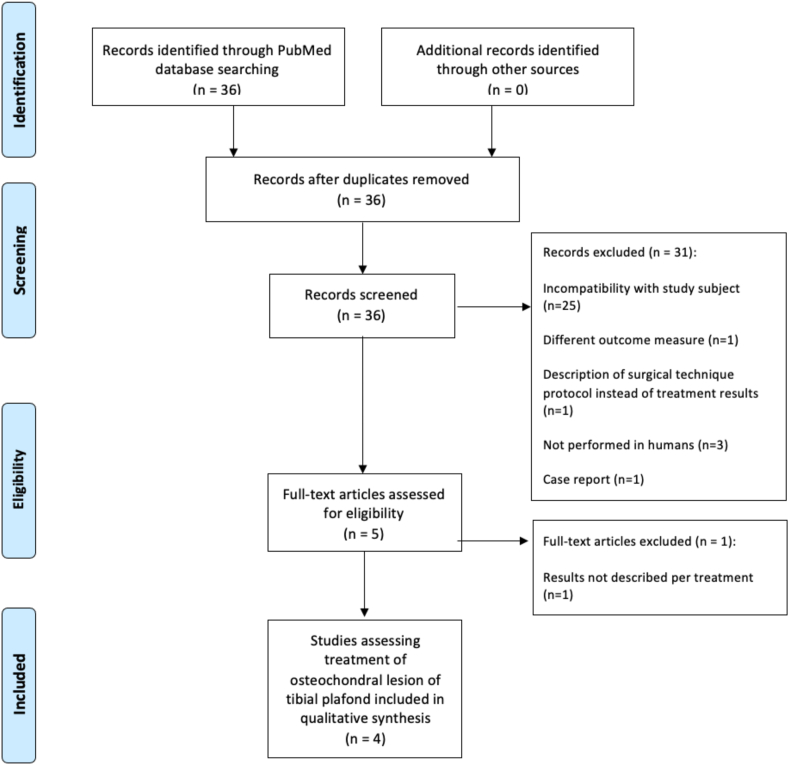
Study selection described in the PRISMA flowchart.

In the PubMed/MEDLINE database, 36 articles were identified of which titles and abstracts were screened. A total of 31 articles were excluded due to incompatibility with study subject (e.g. pilon/knee/malleolar fractures or knee instability (n = 16), treatment of talar osteochondral lesions (n = 9), description of incidence of OLTPs (n = 1), description of a surgical technique protocol for OLTPs (n = 1)). Further reasons for exclusion were that the study was not performed in humans (n = 3) or it comprised a case report (n = 1). Five publications describing the results of treatment of OLTPs were identified and full texts were analyzed regarding eligibility, one of which was subsequently excluded because results were not described per treatment. No relevant studies were identified in the Cochrane Database of Systematic Reviews or Google Scholar, leading to four studies included in this systematic review. The process of study selection is depicted in Fig. 1.

All studies, one Italian^19^ and three American,^4^^,^^10^^,^^20^ concerned retrospective case series. Three of them were classified as therapeutic level IV studies,^4^^,^^10^^,^^20^ while the level of evidence was not mentioned in one study.^19^ Three studies described the results of arthroscopic microfracture treatment,^4^^,^^10^^,^^20^ of which one also described antegrade drilling,^4^ and one describing results of arthroscopically one-step bone marrow-derived cell transplantation (BMDCT).^19^ An overview of the study characteristics and patients is shown in Table 1.

**Table 1:** Characteristics of the included studies and patients.

Author	Year	Country	Design	Subjects (N)	Study population characteristics	In- and exclusion criteria	Type of lesion	Lesion size Mean (±SD)	Location of lesion	Medical ankle history	Treatment	Follow-up Mean
Lee et al.^10^	2019	U.S.A.	Retro-spective case series	16	Mean age 42.1 years (range 18–64), male 31.3%	*Inclusion:* 1-year follow-up available, OLTP confirmed with MRI^a^ *Exclusion:* patients with ankle fracture, no informed consent, lost to follow-up	Isolated OLTP 75.0% (N = 12) Bipolar lesion 25.0% (N = 4) Kissing lesion 0.0% (N = 0)	65.2 mm^2^ (±43.2 mm^2^)	Medial 37.5% (N = 6) Central 43.8% (N = 7) Lateral 18.8% (N = 3)	Trauma 37.5% (N = 6) Unknown 62.5% (N = 10)	Arthroscopic microfracture (N = 16)	30 months
Baldassarri et al.^19^	2017	Italy	Retro-spective case series	27	Mean age 39.2 years (range 19–49), male 55.6%	*Inclusion:* patients 18-15 years suffering various ankle chronic symptoms including pain, stiffness, swelling and locking with a grade III-IV OLTP (ICRS classification)^b^ *Exclusion:* patients with severe osteoarthritis, rheumatoid or haemophilic arthritis, presence of kissing lesion	Isolated OLTP 100.0% (N = 27) Bipolar lesion 0.0% (N = 0) Kissing lesion 0.0% (N = 0)	180 mm^2^ (±47 mm^2^)	Medial 59.3% (N = 16) Central 29.6% (N = 8) Lateral 11.1% (N = 3)	Trauma 74.1% (N = 20) Unknown 25.9% (N = 7)	One-step bone marrow-derived cell transplantation (N = 27)	72 months
Ross et al.^20^	2014	U.S.A.	Retro-spective case series	31	Mean age 37.0 years (range 15–68), male 48.0%	*Inclusion: -* *Exclusion:* follow-up < 24 months	Isolated OLTP 54.8% (N = 17) Bipolar lesion 6.5% (N = 2) Kissing lesion 38.7% (N = 12)	38 mm^2^	Medial 51.6% (N = 16) Central 32.3% (N = 10) Lateral 16.1% (N = 5) Left ankle 45.2% (N = 14) Right ankle 54.8% (N = 17)	Unknown 100.0% (N = 31)	Arthroscopic microfracture (N = 31)	44 months
Cuttica et al.^4^	2012	U.S.A.	Retro-spective case series	13	Mean age 33.0 years (range 14–49), male 69.0%	*Inclusion:* patients failed initial conservative care *Exclusion:* patients who underwent treatment by other than arthroscopic means, who displayed radiographic signs of arthritis, or follow-up < 6 months	Isolated OLTP 69.2% (N = 9) Bipolar lesion 23.1% (N = 3) Kissing lesion 7.7% (N = 1)	>100 mm^2^ (N = 6) <100 mm^2^ (N = 7)	Medial 30.8% (N = 4) Central 38.5% (N = 5) Lateral 30.8% (N = 4) Left ankle 46.2% (N = 6) Right ankle 53.8% (N = 7)	Trauma 46.2% (N = 6) Chronic instability 23.1% (N = 3) Unknown 30.8% (N = 4)	Arthroscopic microfracture (N = 11) Antegrade drilling (N = 2)	39 months

a MRI: Magnetic Resonance Imagine.

b ICRS classification: The International Cartilage Repair Society Cartilage Lesion Classification System.

### 3.2 Quality assessment of included studies

The adjusted NOS^17^ scores of the included studies are summarized in Table 2.

**Table 2:** Summary of quality assessment of included studies with help of the adjusted NOS.

Case series	Year	Study design	Selection	Outcome	NOS Score
Baldassarri et al.^19^	2017	*	*		2
Cuttica et al.^4^	2012	*	*		2
Lee et al.^10^	2019	*	*		2
Ross et al.^20^	2014	*	*		2

### 3.3 Patient characteristics

Eighty-seven patients with OLTPs were included in the studies, the number per study ranging from 13 to 31. Patients' age ranged between 14 and 68 years, and the OLTPs were incurred predominantly by males (range 31.3–69.0%). Information about the side of the ankle injury was available in two studies (N = 44) (right ankle: 54.5%). Mean OLTP size ranged from 38 to 180 mm^2^ (N = 74), and, according to the classification of Elias et al.,^6^ 48.3% of the OLTPs were localized medially, 34.5% centrally and 17.2% laterally (N = 87). Information on the mechanism of injury was available in three studies (N = 56), reporting a trauma in 57.1%, chronic ankle instability in 5.4%, and non-traumatic or not reported causes in 37.5%. Lesions (N = 87) concerned an isolated OLTP in 74.7% of cases, a bipolar lesion (OLT and OLTP concurrently) in 10.3%, and a kissing lesion (OCLs contacted with each other) in 14.9%. The majority of studies used the MOCART^21^ and the AOFAS^22^ scoring systems. Study characteristics are presented in Table 1.

### 3.4 Treatment strategies

The mean follow-up of the studies was 46.2 months (range; 30–72). Three different treatments (arthroscopic microfracture treatment, antegrade drilling and BMDCT) and six different outcome scores (AOFAS, VAS, FAAM, SF-12, FAOS, MOCART) were used. The treatment strategies and their outcomes are presented in Table 3, Table 4.

**Table 3:** Healing of cartilage per treatment strategy.

Treatment	Study	Patients (N)	Reporting system	Complete defect infill in %	Subchondral edema or cyst in %
BMDCT^a^	Baldassarri et al.^19^	27	MOCART^b^	68.0	28.0
Microfracture	Ross et al.^20^	23	MOCART	52.0	65.0

a BMDCT: One-step Bone Marrow-Derived Cell transplantation.

b MOCART: Magnetic Resonance Observation of Cartilage Repair Tissue.

**Table 4:** Function level scores per treatment strategy.

Treatment	Study	Patients (N)	Reporting system	Pre Mean (range)	Post Mean (range)	Difference	P-value
Antegrade drilling	Cuttica et al.^4^	2	AOFAS^a^	28.5 (24–33)	44 (33–55)	+15.5	–
BMDCT^b^	Baldassarri et al.^19^	27	AOFAS	52.4	80.6	+28.2	–
Microfracture	Cuttica et al.^4^	11	AOFAS	36.8 (28–49)	51.3 (42–55)	+13.8	–
	Lee et al.^10^	16	VAS^c^	8.3 (6–10)	1.8 (0–4)	+6.5	**<0.00**
			FAAM ADL^d^	57.6 (6.0–88.9)	84.3 (46.4–100.0)	+26.7	**<0.00**
			FAAM Sports^e^	34.5 (3.1–92.6)	65.2 (23.3–55.1)	+30.7	**<0.00**
			SF-12 PCS^f^	36.3 (23.3–55.1)	46.0 (18.9–56.6)	+9.7	**0.00**
	Ross et al.^20^	31	FAOS^g^	50.5 (17–75)	74.2 (47–92)	+23.7	**<0.01**
			SF-12^h^	38.7 (3–57)	59.5 (16–89)	+20.8	**<0.01**

a AOFAS: American Orthopaedic Foot and Ankle Society score.

b BMDCT: One-step Bone Marrow-Derived Cell transplantation.

c VAS: Visual Analogue Scale.

d FAAM ADL: Foot and Ankle Ability Measure Activity daily living subscale.

e FAAM Sports: Foot and Ankle Ability Measure Sports subscale.

f SF-12 PCS: Short-form generic measure of health status, Physical Component Summary.

g FAOS: Foot and Ankle Outcome Score.

h SF-12: Short-form generic measure of health status.

#### 3.4.1 Antegrade (malleolar) drilling

If the cartilage cap of the OLTP was intact, the defect could be drilled through the malleolus. However, microfracture treatment was favored over drilling, because of the difficulty to make the microfracture holes perpendicular to the subchondral plate and the risk of thermal necrosis with drilling.^4^ Cuttica et al.^4^ described the results of the antegrade drilling treatment in two patients. No description of the effect on healing of cartilage was available. In one patient the AOFAS score improved from 24 preoperatively to 55 after surgery, while in one patient no improvement in the AOFAS score was observed after surgery.

#### 3.4.2 One-step bone marrow-derived cell transplantation (BMDCT)

BMDCT comprises a biological reconstructive technique, aimed at the restoration of a layer of cartilage as similar as possible to hyaline cartilage. The technique consists of a few phases including platelet gel production, bone marrow aspiration from the posterior superior iliac crest followed by concentration of this bone marrow and the surgical procedure to transplant it to the defect site on the tibial plafond. Baldassarri et al.^19^ described the results of this treatment for N = 27 patients. In that study, MRI showed a complete osteochondral defect filling in 68.0% of the patients according to the MOCART score. Furthermore, the mean AOFAS score improved from 52.4 preoperatively to 80.6 at the final follow-up. No complications were observed post-surgery.

#### 3.4.3 Arthroscopic microfracture

In case of arthroscopic microfracture, excision, debridement and curettage of unstable cartilage flaps or fragments, synovectomy and micro fracturing are performed. The micro-fracturing partially destroys the calcified zone that is most often present, and creates multiple openings into the subchondral bone, leading to a release of growth factors and therefore the formation of fibrin clots. Eventually, bone marrow cells are introduced in the osteochondral lesion, and fibro-cartilaginous tissue is formed. Three publications described the results of this treatment for a total of 58 patients.^4^^,^^10^^,^^20^ In the study of Ross et al.,^20^ who followed N = 31 patients, MRI showed complete osteochondral defect filling in 52.0% of patients according to the MOCART score. Additionally, Ross et al.^20^ showed significant improvements in the FAOS and the SF-12 outcome scores. Cuttica et al.,^4^ investigating N = 10 patients who underwent this treatment, reported poor results in two, fair results in two and good results in six patients, established using the AOFAS score. Lee et al.,^10^ which followed N = 16 patients, showed a significant improvement in all function outcome scores i.e. FAAM, SF-12 and VAS.

## 4 Discussion

The aim of this systematic review was to evaluate reports on the effectiveness of surgical treatments on the healing of cartilage and on the function level, in terms of pain reduction, reduced swelling and improved joint range of motion, in patients with osteochondral lesions of the tibial plafond. This review summarized reported outcomes of four studies, totaling 87 patients with osteochondral lesions of the tibial plafond, and describing the effectiveness of 3 treatments. According to the results of the includes studies in this review, arthroscopic treatment of OLTP by means of microfracture and BMDCT seem effective for the outcome at the patient's function level, while BMDCT showed more promising results regarding defect filling compared to arthroscopic treatment by means of microfracture.

All studies that investigated arthroscopic microfracture for the treatment of OLTP showed an overall (significant) improvement in patients' outcome at function level.^4^^,^^10^^,^^20^ For OLTs, arthroscopic microfracture is a widely accepted treatment with good clinical outcomes, and therefore this treatment was expected to have good clinical outcomes in the OLTP population as well.^10^ The BMDCT treatment showed the highest percentage of patients with a complete filling of the osteochondral defect and showed an overall improvement in the patient's outcome at function level.^19^ The antegrade drilling treatment, evaluated in the study of Cuttica et al.,^4^ reported only two cases with contrasting outcomes at the patients' function level outcome.^4^ Moreover, this antegrade drilling treatment is associated with high iatrogenic risks, and therefore only preferred if the cartilage cap is still intact.^4^

These results are in line with the results of the study of Mologne et al.,^5^ which described that arthroscopic treatment by means of curettage, debridement, abrasion arthroplasty, and, in some patients transmalleolar drilling, microfracture of iliac crest bone grafting showed good results in 14 of 17 patients using the AOFAS score. However, this study was not included in the current review since the study did not describe results separately per surgical treatment.

Regarding the healing of cartilage, Ross et al.^20^ showed edema or subchondral cysts in 65.0% of their cases. Correspondingly, Cuttica et al.,^4^ found a correlation between MRI edema and clinical outcomes following microfracture treatment.^23^ Also, Cuttica et al.^4^ showed postoperative bone marrow edema on MRI of all patients with poor outcomes.^4^ In contrast, Baldassari et al.^19^ found edema or subchondral cysts in only 28.0% of cases, suggesting that surgery would have less impact on subchondral bone.

Furthermore, regarding the patients' return to sports after surgery, the study of Lee et al.^10^ described that although all patients were able to return to sports activity after surgery, the postoperative level of sports activity was significantly lower than the preoperative level based on one of the questions of the FAAM score.

The present review represents the first identification of the currently known treatment options for primary OLTPs. Although literature on the treatment of OLTPs is scarce and high evidence level studies are lacking, the present systematic review raises awareness on the subject which may encourage more research on this topic. Treatment recommendations for OLTPs are of paramount importance to achieve the most optimal healing of cartilage and function outcome. The low frequency of OLTPs reported in literature can be questioned, since the study of Lee et al. (2019) reported a ratio of 6.1:1 regarding the frequency of OLTs versus OLTPs.^10^ The study of Irwin et al. (2018) also described that the incidence of coexisting OLTs and OLTPs may be more prevalent than suggested by previous reports, indicating a higher incidence of OLTPs compared to previous literature.^24^ Furthermore, under-diagnosing has been reported in up to 50.0% of cases due to the difficulty in identifying OCLs by conventional radiographs.^24^ This, in turn, leads to a delayed diagnosis or surgery.^5^ Therefore, a follow-up MRI or Computer Tomogram (CT) is necessary in cases involving ankle injury with no resolution at six to eight weeks or with persistent limitations.^9^ In future a more rigorous diagnostic approach should be used to identify these lesions.

### 4.1 Limitations of the present study

This review also faces some limitations. No RCTs or prospective comparative studies were found, and only retrospective case series were available for inclusion, leading to a low level of evidence. Due to the rarity of OLTPs, literature on the treatment of OLTP is very scarce and includes mainly case reports and case series, leading to a small number of studies and patients to be included. All studies lacked controls, had a wide range of follow-up and lesion sizes, a very heterogeneous study population and/or lacked MOCART scores.^4^^,^^10^ Due to this low number of patients, the statistical power is limited, and type-II error might occur. Study (population) characteristics showed too much variability for a reliable interpretation of the results.

Although the NOS adjusted for case series has not been validated, a low score indicates a higher chance of bias. Overall, the quality of the retrospective case series was poor, and all four studies were likely at high risk of bias (100%). On the other hand, this review, for the first time, focusses on the impact of OLTPs and should be regarded as an initiative to start methodologically sound comparative studies.

Due to the paucity of data on clinical outcomes of OLTP treatments and the poor quality of methodology in all four studies included in the review, no conclusions can be drawn yet. In order to compare the outcomes of surgical strategies for OLTPs and to draw definitive conclusions, further studies are necessary, including sufficiently powered, randomized clinical trials with longer follow-up periods and a larger number of cases.

## 5 Conclusions

The results of the included studies in this review showed that treatment by means of microfracture and BMDCT might be promising for the patient's outcome at function level and healing of cartilage. However, no conclusions can be drawn since this is based on the current available evidence with poor quality of methodology due to paucity of good data on the subject. Nevertheless, this review raises awareness on the subject which may encourage more research on this topic. Further research is of paramount importance to understand this injury and to evaluate the best treatments.

## Ethical approval

Not applicable.

## Consent for publication

Not applicable.

## Availability of data and materials

Not applicable.

## Funding

None declared.

## Declaration of competing interest

None declared.
